# Altered movement strategy during functional movement after an ACL injury, despite ACL reconstruction

**DOI:** 10.3389/fspor.2022.994139

**Published:** 2022-10-04

**Authors:** Lauri Stenroth, Cecilie Bartholdy, Jonas Schwarz Larsen, Mads Skipper Sørensen, Kenneth B. Smale, Teresa E. Flaxman, Daniel L. Benoit, Michael R. Krogsgaard, Tine Alkjær

**Affiliations:** ^1^Department of Biomedical Sciences, University of Copenhagen, Copenhagen, Denmark; ^2^Department of Applied Physics, University of Eastern Finland, Kuopio, Finland; ^3^The Parker Institute, Bispebjerg and Frederiksberg Hospital, Copenhagen, Denmark; ^4^School of Human Kinetics, University of Ottawa, Ottawa, ON, Canada; ^5^School of Rehabilitation Sciences, University of Ottawa, Ottawa, ON, Canada; ^6^Section for Sports Traumatology, M51, Bispebjerg and Frederiksberg Hospital, Copenhagen, Denmark

**Keywords:** kinematics, kinetics, movement quality, patient-reported outcomes, muscle strength

## Abstract

Knee joint functional deficits are common after anterior cruciate ligament (ACL) injury, but different assessment methods of joint function seem to provide contradicting information complicating recovery monitoring. We previously reported improved perceived knee function and functional performance (forward lunge ground contact time) in patients with an ACL injury from pre to 10 months post ACL reconstruction without improvement in knee-specific biomechanics. To further investigate this discrepancy, we additionally analyzed knee extensor and flexor muscle strength, and movement quality in the forward lunge (subjective and objective evaluations) and performed a full lower limb biomechanical analysis of the forward lunge movement. We included 12 patients with an ACL injury (tested before and after ACL reconstructive surgery) and 15 healthy controls from the previous study to the current investigation. Outcome measures were obtained pre and ~11 months post ACL reconstruction for the patients and at a single time point for the controls. Objective movement quality in the patients with an ACL injury showed an improvement from their pre reconstruction surgery visit to the post reconstruction visit but this was not observable in the subjective evaluation. Knee extensor muscle strength declined after the ACL reconstruction by 29% (*p* = 0.002) and both knee extensors (*p* < 0.001) and flexors (*p* = 0.027) were weaker in the patients post ACL reconstruction compared to healthy controls. ACL injured patients had an altered movement strategy in the forward lunge with reduced knee extensors contribution and increased hip extensor contribution compared to the controls both before and after the reconstruction. The altered movement strategy was associated with knee extensor muscle strength. This explorative study with a limited sample size found that clinicians should be aware that significant functional deficits in the knee extensor muscles, both in isolated muscle strength testing and during a functional movement, may be present although patients perceive an improvement in their knee function and present good functional performance without obvious movement quality issues.

## Introduction

Anterior cruciate ligament (ACL) injury is a sports-related knee injury that is commonly treated with ACL reconstruction surgery. There is a high risk of long-lasting functional deficits in knee-crossing muscles (1), secondary ACL injuries (2) and an increased risk of development of knee osteoarthritis (3–5) in individuals with an ACL injury. The functional deficits in knee extensor muscles have been suggested to play a role in potential adverse outcomes after the injury (6, 7). Therefore, it is of particular interest to monitor knee function in patients recovering from an ACL injury.

Common clinical tools to monitor knee function include functional performance tests, such as jump tests, and patient-reported outcomes (PROMS) of perceived knee function. In our previous study (8), in which we examined knee function pre and 10 months post ACL reconstruction in a forward lunge task, an improvement in knee function based on PROMS and our primary functional performance outcome (shorter movement time) was observed. However, there were no changes in knee-specific biomechanics, i.e., peak knee extensor moment during the forward lunge. Additionally, the functional performance 10 months post ACL reconstruction was comparable with that of the uninjured controls group despite the peak knee extensor moment during the forward lunge persisted to be significantly lower. This suggests that scores of PROMS and functional performance are not enough to describe knee joint function observed during a functional task and may hide clinically important deficits. They further suggest that the patients may utilize a movement strategy, enabled by compensation from other joints, which allows for overall unchanged functional performance in the presence of persistent knee joint functional deficit. Severe preoperative strength deficit in quadriceps and hamstring muscles has been reported in patients with an ACL injury (9) and a potential reason for the persistent deficit in knee joint function is the potential lack of improvement in muscle strength from pre to post ACL reconstruction.

To elucidate the reasons for the discrepant results arising from PROMS and functional performance test compared to knee joint biomechanical analysis we revisited the data and expanded our analysis to include other lower limb joints in the biomechanical assessment and knee extensor and flexor muscle strength measurements. We additionally analyzed lower limb movement quality (based on subjective observation and objectively measured) since monitoring the movement quality has been suggested as one of the tools to guide progression from one rehabilitation stage to the next after ACL injury (9) and has been found to be associated with the risk of primary and secondary ACL injury (10, 11). Accordingly, the objectives of this study were to (1) compare knee extensor and flexor muscle strength before and after ACL reconstruction and in comparison to healthy controls, (2) compared lower limb joint mechanical output in the forward lunge (peak moment and power and contribution to total joint work) before and after ACL reconstruction and in comparison to healthy controls, (3) investigate if knee extensor muscles strength is associated with movement strategy (contribution of the joint on lower limb mechanical work) observed while performing the forward lunge in ACL injured patients and in healthy controls and (4) investigate changes in movement quality in patients with an ACL injury from pre- to post ACL reconstruction and compare movement quality between patients with an ACL injury and healthy controls. We hypothesized that muscle strength is lower in the patients with an ACL injury compared to healthy controls and does not improve during the follow-up as our previous investigation showed a persistent deficit in the peak knee extensor moment during the forward lunge. We additionally hypothesized that the observed improvement in functional performance (i.e., shorter movement time) is due to increased mechanical output from hip extensors as it has been previously shown that patients with an ACL injury compensate with hip extensors for the lower mechanical output of knee extensors in both horizontal and vertical jumps (12–14). Finally, we hypothesized that patients with an ACL injury have inferior movement quality compared to healthy controls and that the movement quality improves from pre to post ACL reconstruction since the perceived knee function showed an improvement in the previous study in this sample (8).

## Materials and methods

### Participants

This study utilizes data obtained in a previous experiment from which other results have been published (8, 15–20). For this investigation, we selected the participants that had knee extensor and flexor muscle strength tests performed, and if ACL injured, had attended experimental sessions both pre and post ACL reconstruction. This subsample included 12 ACL injured patients and 15 controls (Table 1).

**Table 1:** Participant characteristics.

	**ACLd** **(*n* = 12)**	**ACLr** **(*n* = 12)**	**Control** **(*n* = 15)**
Sex (females/males)	3/9	3/9	6/9
Time since injury (months)	18 ± 28	29 ± 28	N/A
Time since surgery (months)	N/A	11 ± 1	N/A
Age (years)	27 ± 6	28 ± 6	27 ± 9
Height (m)	1.80 ± 0.07	1.80 ± 0.07	1.78 ± 0.08
Mass (kg)	76.6 ± 6.2	76.7 ± 6.5	74.5 ± 15.2
BMI (kg/m^2^)	23.8 ± 2.2	23.8 ± 2.1	23.3 ± 3.7

The participants were recruited among the ACL injured individuals waiting for ACL reconstruction at Bispebjerg and Frederiksberg Hospital (Copenhagen, Denmark). Eligible participants were between 18 and 50 years of age, had a clinically verified ACL tear in one knee (positive Lachman test, positive pivot shift and increased anterior tibial translation measured with Rolimeter and compared to the healthy knee; confirmation during surgery) and a normal contralateral knee, were free of pain in the lower extremities, had no neurological/cardiovascular diseases, and were not pregnant. Healthy matched (based on age, sex, height, and body mass) volunteers were recruited among colleagues and relatives of employees at the University of Copenhagen. Note that the matching was performed on the original sample while a subsample was included in this study. Before participation, participants gave written informed consent for the study. The study was approved by the ethics committee for the Capital Region of Denmark (H-3-2013-126) and the University of Ottawa Ethics Board (H06-14-27) and was performed in accordance with the Helsinki II declaration.

Nine out of the twelve ACL injured participants underwent a doubled hamstring autograft reconstruction procedure, in one participant a bone-patella-bone autograft was used and in one male an Achilles tendon allograft was used. The median time from injury to pre reconstruction study visit was 6 months (range: 1–101 months) and the median time from reconstruction to post reconstruction study visit was 11 months (range: 10–13 months). All patients received a standardized 20-week rehabilitation program that included range of motion, balance, strength, and functional training components, with a progression in the intensity [see details from (21)]. More details on the participants can be found in the Supplementary material.

### Experimental procedures

Patient perceived knee function was assessed by two questionnaires: The Lysholm score (15) and the International Knee Documentation Committee (IKDC) subjective form (16). The intensity of physical activities performed by the patients was assessed with the Tegner score (17). The questionnaires were completed during the pre and post reconstruction laboratory visits. The patients were additionally asked to report pre injury Tegner scores. A single patient had a missing score from the Lysholm and Tegner questionnaires pre-surgery and two participants were missing the pre injury Tegner score.

Maximal voluntary isometric contraction (MVIC) moment for knee flexion and extension were measured using an isokinetic dynamometer (KinCom, Kinetic Communicator, Chattecx Corp., Chattanooga, USA) operated at isometric mode. Participants were tightly secured to the dynamometer, in a seated posture, and the knee joint axis was carefully aligned with the dynamometer axis. The moment measured during the MVIC testing was gravity corrected. The moment created by gravitational forces on the leg was measured for each subject at 40 degrees knee flexion, whereas the MVIC was measured at 30 degrees knee flexion for both the quadriceps and the hamstrings. The difference in the joint angle between the measurement of the gravitational moment and MVIC was due to other tests performed in parallel to this investigation. However, the experimental setup was consistent throughout the experiment and should not bias our group comparisons or investigated associations. The patients performed three trials to assess knee extension muscle strength followed by three trials to assess knee flexion muscle strength both with the operated leg. The test leg for the healthy controls was based on matching the distribution of dominant legs tested in the ACL injured group. MVIC was defined as the peak moment observed across the three trials for each exercise.

Forward lunge was performed in a motion capture laboratory. The instruction for the participants was to perform forward lunge movements at a self-selected pace by taking one step forward, placing the foot on the force plate, flexing the knee to ~90° and subsequently pushing themselves back into the starting position, while having their hands on the back of their head, the upper body perpendicular to the ground, and the opposite foot maintaining contact with the ground. The lunge movement was performed with their hands at the back of the head to control upper body motion and standardize the movement between participants. While this approach differs somewhat from unrestricted movements seen in sport, it is a reliable method (18) that is sensitive to the differences in knee extensor muscle function between ACL injured copers and non-copers (19). Given this, we consider this approach suitable for tracking relevant changes in lower extremity biomechanics pre and post operatively. Verbal feedback was provided by the research team if the forward lunge was deemed inadequate (failing to perform the movement as described above) and the repetition was repeated. The patients performed three trials with the operated leg as the leading leg. The test leg for the healthy controls was based on matching the distribution of dominant legs tested in the ACL injured group. Participants were fitted with an extended Plug-in-Gait lower body marker set with a total of 20 markers placed at the pelvis and lower extremities. The markers added to the Plug-in-Gait lower body marker set were markers over the medial epicondyles of the knee and medial malleoli. The movement was recorded with a 10-camera motion capture system (6 MX and 4 T series, Vicon, Nexus, v1.8.5, Oxford Metrics, Oxford, UK) with cameras operating at 100 Hz while ground reaction forces of the leading leg were recorded at 1,000 Hz (OR 6-5-1, AMTI, USA).

### Data analysis and reduction

Kinematic and kinetic analysis was performed using musculoskeletal modeling and simulation software OpenSim (version 4.1, RRID:SCR_002683) (20). Data processing was performed in MATLAB (version R2019b, RRID:SCR_001622). First, a generic musculoskeletal model (22) was scaled to match the dimension of the participant using experimental markers located at specific anatomical landmarks. Segment masses and inertia properties were scaled according to the body mass of the participant. Subtalar and metatarsal joints were kept locked to an anatomically neutral position during the analyses. The resulting model had three degrees of freedom (DoF) for the hip joints and one DoF for the knee and ankle joints. Then, the kinematics were estimated using OpenSim's inverse kinematics tool that finds values for generalized coordinates (joint angles and location of the base segment) by minimizing the sum of squared differences between experimental and model marker locations at each time instant of the data. Finally, joint kinetics were calculated using OpenSim's inverse dynamics tool. Ground reaction forces and joint kinematics were filtered using a matching zero-lag 4^th^ order Butterworth low-pass filter with a 15 Hz cut-off frequency (23) before entering the inverse dynamics and joint power calculations. The analyses in OpenSim were performed only for the period of ground contact made with the leading leg which was defined using a 25 N threshold for the vertical ground reaction force. The duration of this period was defined as the lunge movement time.

Joint powers were calculated as the dot product between the time derivative of joint angles (joint angular velocities) and joint moments. Joint work was calculated by integrating the joint power over time. This was done separately for negative and positive power phases to calculate the amounts of negative (eccentric work at the level of the muscle-tendon unit) and positive (concentric work at the level of the muscle-tendon unit) work performed by the muscles crossing each joint.

The outcomes extracted from the biomechanical assessment of the lunge were peak knee flexion angle, peak knee extensor moment, peak negative and positive knee joint power and the contribution of work performed at the ankle, knee, and hip joints to the total negative and positive work. The contribution of each joint to the total work was calculated as:


$$Joint\;contribution\;(\%)=\frac{W_{joint}}{(W_{hip}+W_{knee}+W_{ankle})\;}*\;100,$$

where W_joint_ is the work performed by the joint of interest and W_hip_, W_knee_ and W_ankle_ are the work performed by hip, knee and ankle, respectively. All outcome variables were extracted from each trial after which the outcomes were averaged within a participant before entering the statistical analysis. For graphical representations of the joint kinematics and kinetics, the time series data were first interpolated to 101 data points (0–100 % of stance) and then averaged within the participant.

Motion capture-based kinematic data were used for assessing the lower limb movement quality in two ways. An objective quantitative analysis by calculating the following variables from the kinematic data: knee “wobble,” functional knee alignment, pelvic drop and lateral hip movement. The knee “wobble” was defined as the number of times the knee joint center mediolateral movement path changed direction. Functional knee alignment was quantified as the frontal plane distance of the knee joint center from the line between the hip and ankle joint centers. The pelvic drop was the peak pelvis frontal plane angle with a positive angle resulting from orientation in which the leading leg's hip joint center was higher than the trailing leg. The lateral hip movement was quantified as the lateral movement amplitude of the lead leg's hip joint center. All variables were quantified during the period from ground contact to peak knee flexion. A subjective assessment of the lunge quality was performed by a clinician with 11 years of experience as a physiotherapist of which 2 years focused on treating patients with an ACL injury (C.B.). The assessment was done in random order blinded by group (patients with an ACL injury or healthy controls) and time point (pre- or post-surgery). The assessments were performed on visualizations created based on the motion capture data allowing for the blinding of the clinician in terms of contextual factors such as clothing, body composition, gender, age, and facial expressions. The visualizations were created in OpenSim based on the analyzed kinematics of the participants, using an unscaled generic skeletal model of the lower extremities and pelvis to focus the assessment on the movement of interest. The movement was displayed in sagittal and frontal planes, once at normal speed and once slowed to 20 % of the normal speed. An example of the visualization can be found in the Supplementary material. Based on watching the animation of each of the three trials the clinician evaluated the overall movement quality (composite of all trials) as good or bad based on whether the movement quality raises concern regarding the potential increased risk of a knee injury. Further, the movement quality was subdivided into a four-level scale (good, fairly good, fairly bad, bad). In addition, it was noted if the following movement traits were visible: knee varus, knee valgus, knee side-to-side movement, pelvis side movement or pelvic drop.

### Statistical analysis

Statistical analysis was performed in Jamovi (version 1.8.1) (24). The normality of data was checked using the Shapiro-Wilk test. Comparisons between the pre- and post-surgery time points within the patients with an ACL injury (i.e., ACLd vs. ACLr) were performed using paired samples *t*-test. If the data normality assumption was not met, the Wilcoxon rank sum test was used. ACL injured patients were compared to the control group (i.e., ACLd vs. Control, ACLr vs. Control) using independent samples *t*-test or the Mann-Whitney U test depending on data distribution. The threshold for statistical significance was set at *p* < 0.05. Due to a limited number of participants, we refrain from statistical analysis of the clinically evaluated lower limb movement with categorical variables. Instead, we qualitatively describe this outcome. The association between knee extensor muscle strength and movement strategy (contribution of the joint on lower limb mechanical work) was tested using The Pearson correlation coefficient in Matlab (R2019b, MathWorks, Natick, MA, USA).

The current study is a secondary analysis of an existing dataset (8). The sample size could not be planned based on power calculations but was as large as possible. *Post-hoc* statistical power calculation showed that the sample was sufficiently sized to detect effect sizes with a magnitude of *d* > 0.88 for the within patient groups tests (two-tailed paired *t*-test, α = 0.05, power = 0.8, *N* = 12) and *d* > 1.12 for the tests between patients and controls (two-tailed independent samples *t*-test, α = 0.05, power = 0.8, *N* = 12/15). These are considered large effect sizes and correspond to effects observed in the previous investigation between the patients and controls in peak knee joint moment during FL (8). We considered that for the results to have clinically relevant meaning the effects sizes should be at least large in magnitude. For example, with an effect of *d* = 1, there would be a 62% overlap with the distribution of the two populations meaning that the use of such measures for monitoring a patient should be done with caution.

## Results

### Perceived knee function and physical activities performed by the patients

**Figure 1:**
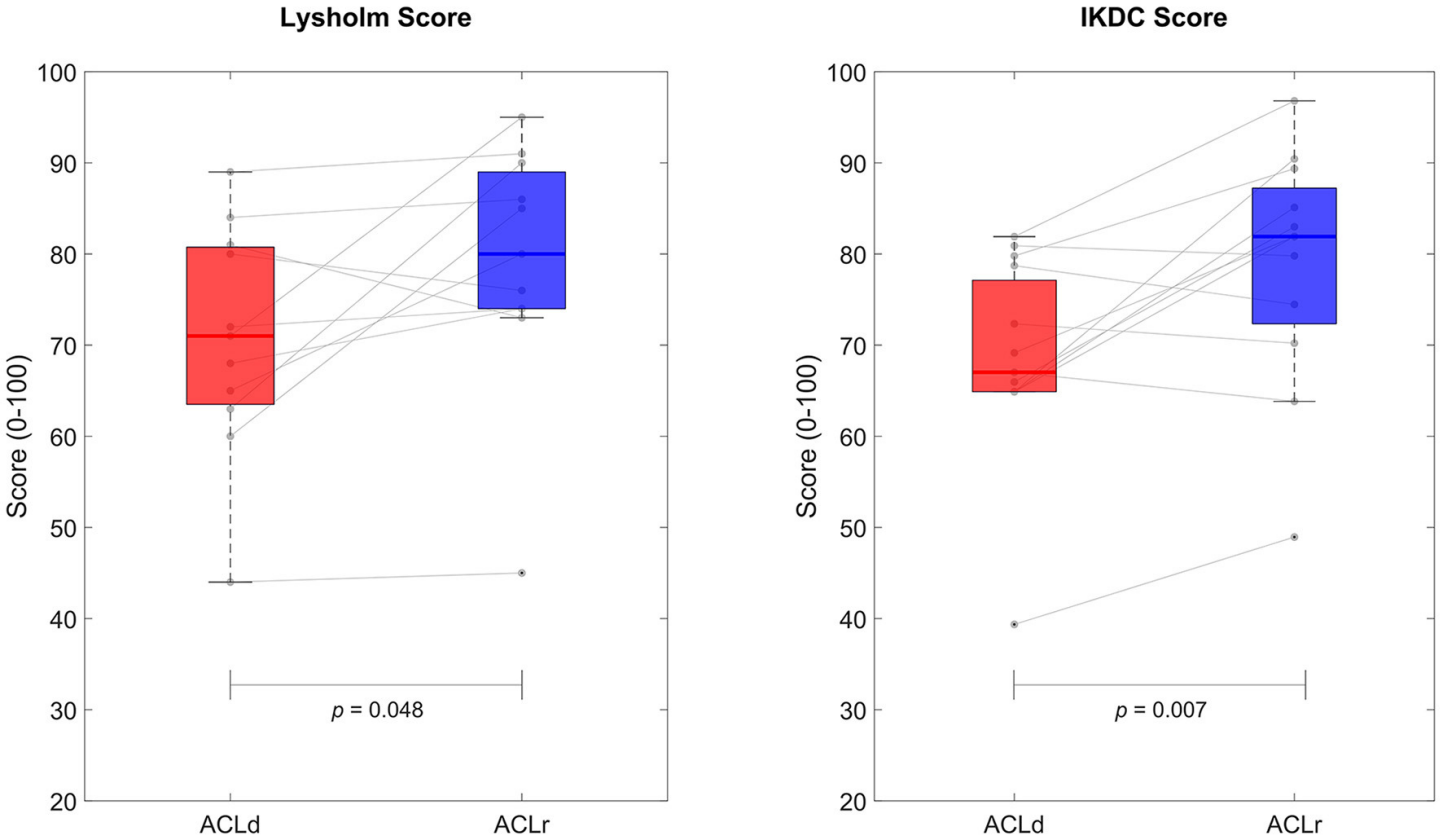
Perceived knee function based on Lyshold and International Knee Documentation Committee (IKDC) scores. Both measures showed statistically significant improvement from pre to post ACL reconstruction.

Both IKDC and Lysholm scores increased from pre to post surgery in the patients with an ACL injury (Figure 1). The mean increase in Lysholm score was 8.4 points [95% CI (0.1, 16.6), *p* = 0.048, *d* = 0.68] and the mean increase in IKDC score was 9.7 points [95% CI (3.2−, 6.1), *p* = 0.007, *d* = 0.96]. The average pre injury Tegner score was 7.7 ranging from 6 to 10 indicating that all patients performed at least recreational sporting activities before the injury (see details from the Supplementary material). The average pre surgery Tegner score was 4.1 (range: 1–10). Tegner score increased from pre to post surgery in the patients on average by 1.5 points [95% CI (0.4, 2.6), *p* = 0.015, *d* = 0.89].

### Knee extensor and flexor muscle strength

**Figure 2:**
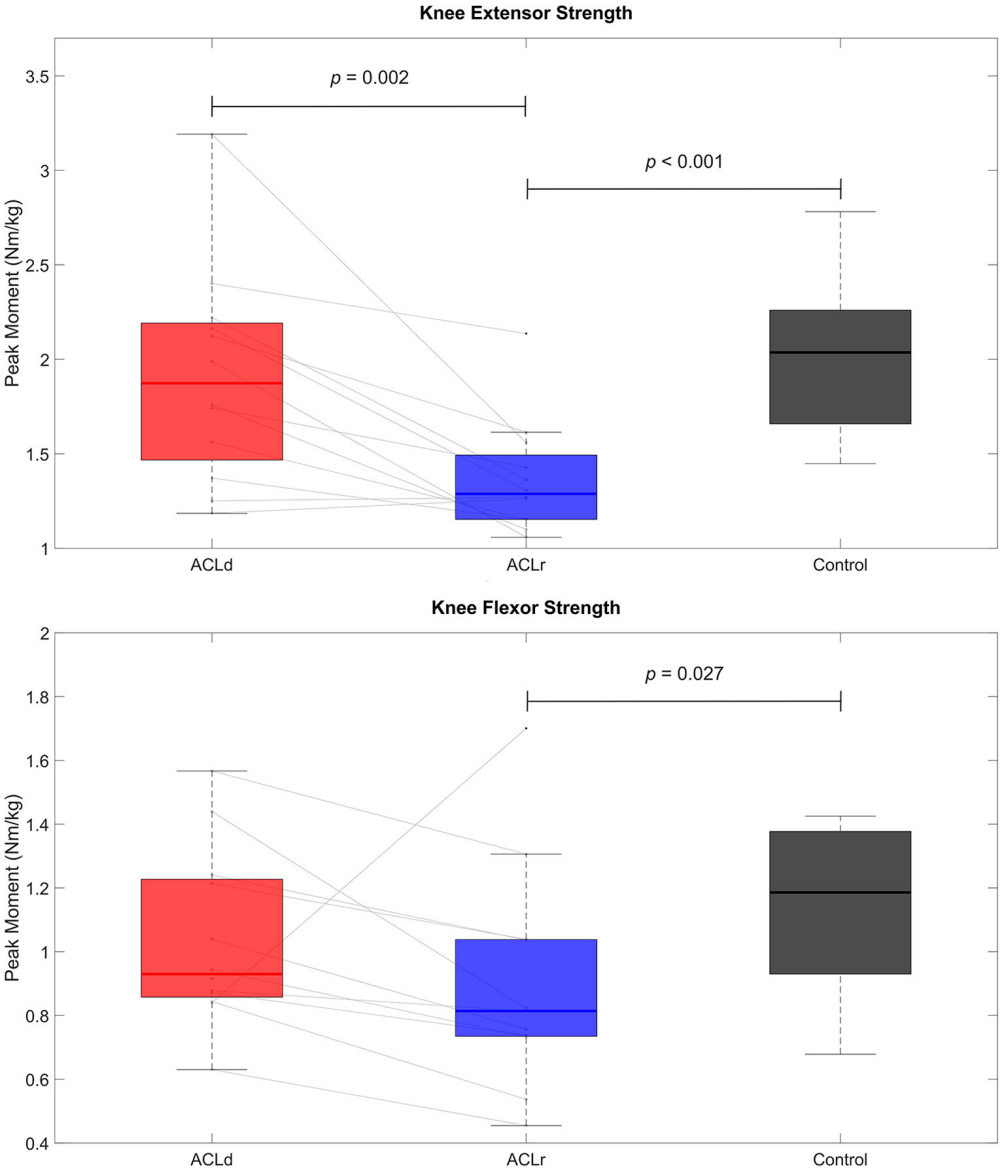
Knee extensor and flexor muscle strength. Knee extensor strength showed a statistically significant decline from pre to post ACL reconstruction. The patients with an ACL reconstruction were weaker in both knee extensors and flexors than healthy controls.

In the patients with an ACL injury, the knee extensor muscle strength of the injured leg decreased by 29% [mean difference −0.55 Nm/kg, 95% CI (−0.85, −0.24), *p* = 0.002, *d* = −1.15] while no significant change in knee flexor strength was observed [mean difference −0.14 Nm/kg, 95% CI (−0.39, 0.10), *p* = 0.054, *d* = −0.39] from pre to post ACL reconstruction. No difference was observed between ACLd and Control in knee extensor [mean difference −0.06 Nm/kg, 95% CI (−0.44, 0.31), *p* = 0.727, *d* = −0.14] or flexor strength [mean difference −0.11 Nm/kg, 95% CI (−0.33, 0.10), *p* = 0.347, *d* = −0.41]. However, the ACLr group was weaker than Control in both knee extension [mean difference −0.61 Nm/kg, 95% CI (−0.88, −0.34), *p* < 0.001, *d* = −1.79] and knee flexion [mean difference −0.24 Nm/kg, 95% CI (−0.49, 0.01), *p* = 0.027, *d* = −0.80, Figure 2].

### Subjective assessment of movement quality

Based on the clinician's evaluation, approximately half of the participants had some issues in their movement quality, but no clear differences were observed in the frequency of observed movement quality issues (movement pattern raising concern regarding the potential increased risk of a knee injury) between the groups. In the four-category scale (good, fairly good, fairly bad, bad) 1/15 (7%) of the Controls were identified as having bad movement quality whereas 3/12 (25%) of both ACLd and ACLr groups were identified to belong to this category. Knee valgus was observed approximately in 1/3 of all participants. Knee side-to-side movement was frequently observed in all groups while pelvis side movement was less frequent. The pelvic drop was observed between 1/5 and 1/2 of the participants. Detailed results can be found in the Supplementary material.

### Objective assessment of movement quality

**Figure 3:**
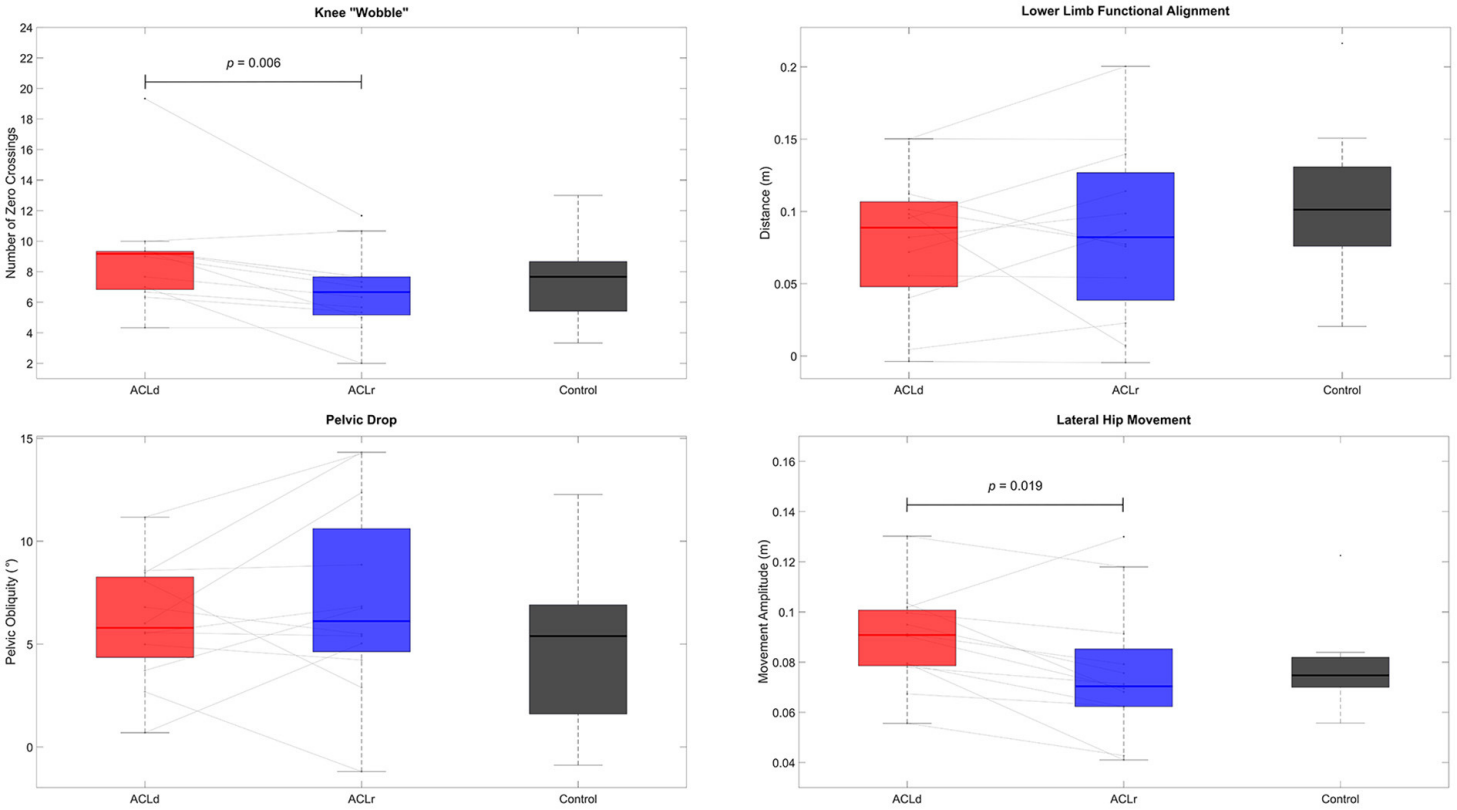
Objective assessment of movement quality. Knee “wobble”, that is describing the mediolateral movement of the knee during the forward lunge, and lateral hip movement reduced from pre to post ACL reconstruction. None of the measures of movement quality differed between the patients with an ACL injury and healthy controls.

Two of the four variables describing the movement quality (knee “wobble,” knee alignment, pelvic drop and lateral hip movement) showed statistically significant improvement from ACLd to ACLr. The knee “wobble” decreased by 26% [mean difference −2.25, 95% CI (−3.73, −0.77), *p* = 0.006, *d* = −0.96] and the lateral hip movement decreased by 22% [mean difference −13.3 mm, 95% CI (−24.0, −2.7), *p* = 0.019, *d* = −0.80]. None of the variables showed statistically significant differences between ACL injured patients and healthy controls either pre or post reconstruction (Figure 3).

### Lower limb joint mechanics during the forward lunge

The ground contact time of the lunge shortened by 25% [mean difference −0.56 s, 95% CI (−0.90, −0.22), *p* = 0.004, *d* = −1.05] from ACLd to ACLr. The time was 35% longer for the ACLd compared to Control [mean difference 0.59 s, 95% CI (0.10, 1.08), *p* = 0.025, *d* = 0.92] but no difference was observed between the ACLr and Control [mean difference 0.03 s, 95% CI (−0.37, 0.42), *p* = 1.000, *d* = 0.05, Supplementary Figure 1].

**Figure 4:**
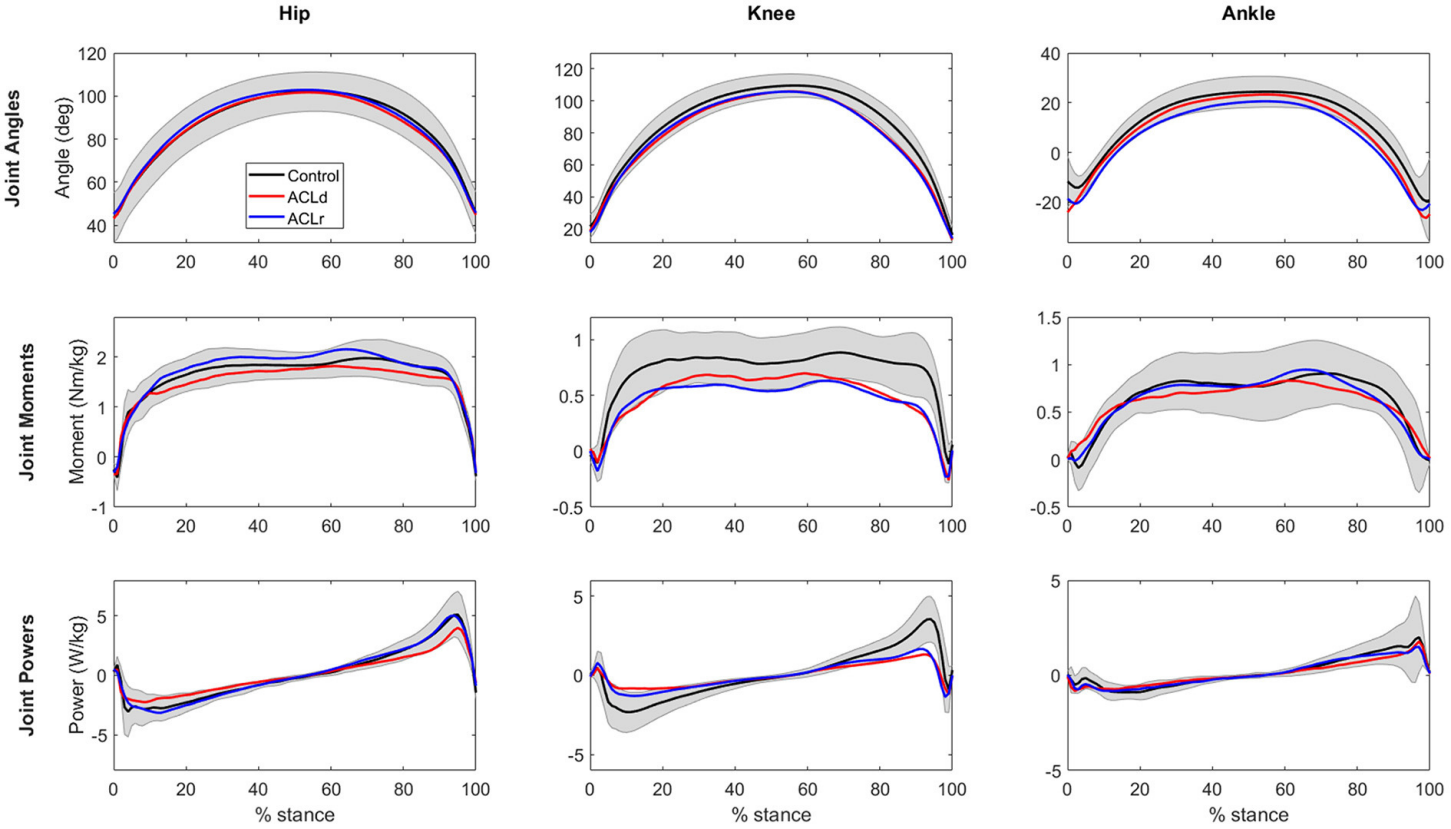
Hip, knee and ankle joint sagittal plane angles and moments and joint power in the stance phase of the forward lunge. For statistical comparison between the groups refer to Table 2. The shaded are represents standard deviation for Control group. Standard deviations for the other groups are omitted for clarity.

**Figure 5:**
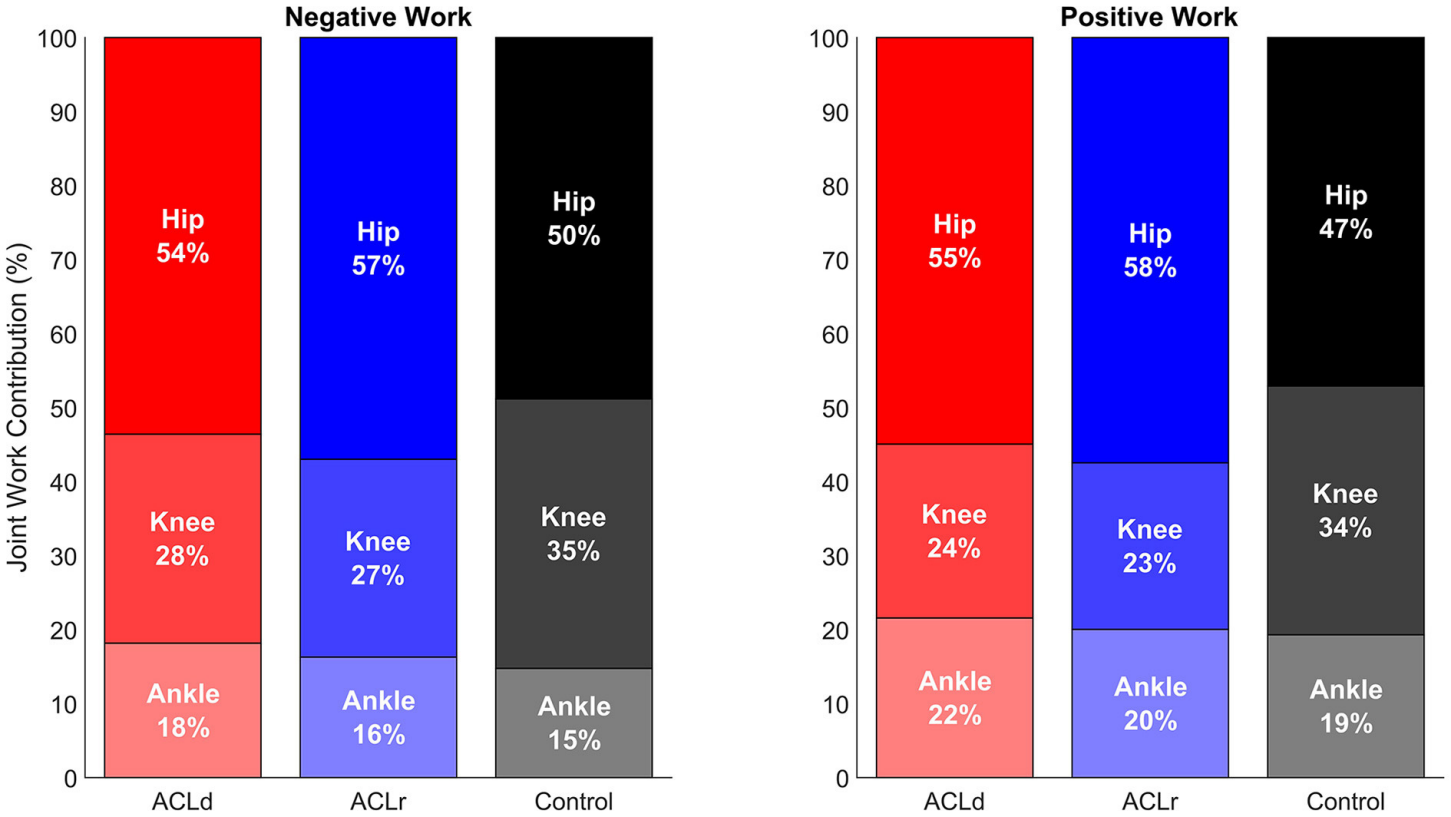
Joint contributions to the lower limb work performed during the forward lunge. The patients with an ACL injury had a statistically significantly lower knee joint contribution on both negative (eccentric) and positive (concentric) work performed compared to healthy controls both pre and post ACL reconstruction. The patients compensated by increasing the contribution of the hip joint which reached a statistically significant difference compared to healthy controls post ACL reconstruction. For details of the statistical comparison between the groups refer to Table 3.

No significant differences in the peak flexion angles of the hip, knee or ankle were observed within the patients with an ACL injury between the two time points or between the patients with an ACL injury and healthy controls (Figure 4; Table 2). Peak hip extensor moment was increased by 15% from ACLd to ACLr while no difference was observed compared to Control at either time point. Peak knee extensor moment did not show a change from ACLd to ACLr but was significantly lower at both time points compared to Control, by 22 and 30%, respectively. ACLr also showed a 28% lower peak ankle plantar flexor moment compared to Control. Peak positive hip extensor power increased by 29% from ACLd to ACLr. Peak negative and positive knee extensor powers were lower in ACLd compared to Control, by 40 and 49%, respectively. Additionally, peak positive knee extensor power was 46% lower in ACLr compared to Control. Knee joint contribution to work performed during the forward lunge was lower in patients with an ACL injury compared to healthy controls at both time points and for both negative and positive work. Conversely, the hip joint contribution was greater in ACLr compared to Control for both negative and positive work (Figure 5; Table 3).

**Table 2:** Peak values of hip, knee and ankle kinematics and kinetics during the forward lunge in patients with an ACL injury and healthy controls.

	**Mean** ±**SD**			**Mean difference [95% CI]**		
				***p*** **-value, effect size**		
	**ACLd**	**ACLr**	**Control**	**ACLd vs. ACLr**	**ACLd vs. Control**	**ACLr vs. Control**
**Peak flexion angles (°)**					
Hip	102.0 ± 7.5	103.0 ± 7.9	102.3 ± 9.1	1.1 [−4.9, 7.0] *p* = 0.702, *d* = 0.11	−0.3 [−7.1, 6.4] *p* = 0.923, *d* = −0.04	0.7 [−6.1, 7.6] *p* = 0.825, *d* = 0.09
Knee	106.5 ± 8.0	106.2 ± 11.4	110.3 ± 6.9	−0.4 [5.1, 4.4] *p* = 0.873, *d* =−0.05	−3.7 [−9.7, 2.2] *p* = 0.206, *d* = −0.50	−4.1 [−11.4, 3.2] *p* = 0.261, *d* =−0.45
Ankle	23.7 ± 9.1	20.7 ± 7.4	24.9 ± 6.0	−2.9 [−6.3, 0.4] *p* = 0.079, *d* =−0.56	−1.2 [−7.2, 4.8] *p* = 1.000^a^, *d* = −0.16	−4.2 [−9.4, 1.1] *p* = 0.118, *d* =−0.63
**Peak extensor moments (Nm/kg)**					
Hip	2.0 ± 0.3	2.3 ± 0.3	2.1 ± 0.3	**0.3 [0.2, 0.4]** ***p*** **<** **0.001**, ***d*** **= 1.36**	−0.1 [−0.4, 0.1] *p* = 0.359, *d* = −0.36	0.2 [−0.1, 0.4] *p* = 0.164, *d* = 0.56
Knee	0.8 ± 0.3	0.7 ± 0.2	1.1 ± 0.1	0.1 [−0.2, 0.0] *p* = 0.186, *d* =−0.41	**−0.2 [−0.4, −0.1]** ***p*** **= 0.011**, ***d*** **= −1.06**	**−0.3 [−0.5,−0.2]** ***p*** **< 0.001**, ***d*** **=−1.6**
Ankle	0.9 ± 0.3	0.7 ± 0.2	1.0 ± 0.3	−0.2 [−0.4, 0.0] *p* = 0.067, *d* =−0.59	−0.1 [−0.3, 0.2] *p* = 0.462, *d* = −0.29	**−0.3 [−0.5,−0.1]** ***p*** **= 0.010**, ***d*** **=1.08**
**Peak positive extensor power (W/kg)**					
Hip	4.4 ± 1.5	5.6 ± 1.4	5.5 ± 1.8	**1.2 [0.7, 1.8]** ***p*** **< 0.001**, ***d*** **= 1.36**	−1.1 [−2.5, 0.2] *p* = 0.093, *d* = −0.68	0.1 [−1.2, 1.4] *p* = 0.879, *d* = 0.06
Knee	2.0 ± 1.3	2.1 ± 1.6	3.9 ± 1.4	0.1 [−0.4, 0.7] *p* = 0.519^a^, *d* = 0.12	**−1.9 [−3.0**, **−0.8]** ***p*** **= 0.001**, ***d*** **= −1.42**	**−1.8 [−3.0,−0.6]** ***p*** **= 0.005**, ***d*** **=−1.19**
Ankle	2.1 ± 1.5	2.2 ± 1.1	2.6 ± 1.8	0.0 [−1.1, 1.1] *p* = 0.992, *d* = 0.00	−0.5 [−1.8, 0.8] *p* = 0.486^a^, *d* = −0.30	−0.5 [−1.7, 0.7] *p* = 0.719^a^, *d* =−0.32
**Peak negative extensor power (W/kg)**					
Hip	−3.1 ± 1.5	−3.5 ± 0.9	−3.8 ± 1.9	−0.4 [−1.1, 0.3] *p* = 0.259, *d* =−0.34	0.5 [−0.6, 2.1] *p* = 0.256^a^, *d* = 0.43	0.4 [−0.9, 1.6] *p* = 0.867^a^, *d* = 0.22
Knee	−1.8 ± 0.8	−2.3 ± 1.4	−3.0 ± 1.3	−0.5 [−1.5, 0.4] *p* = 0.339^a^, *d* =−0.36	**1.2 [0.3, 2.1]** ***p*** **= 0.010**, ***d*** **= 1.09**	0.7 [−0.4, 1.7] *p* = 0.228, *d* = 0.48
Ankle	−1.3 ± 0.6	−1.2 ± 0.5	−1.3 ± 0.4	0.1 [−0.5, 0.6] *p* = 0.774, *d* = 0.08	0.0 [−0.4, 0.4] *p* = 0.989, *d* = 0.01	0.1 [−0.3, 0.4] *p* = 0.663, *d* = 0.17

a P-value from a non-parametric test.

The bold values indicate the comparisons with statistically significant differences between the groups.

**Table 3:** Joint contributions to total work performed during the forward lunge in patients with an ACL injury and healthy controls.

	**Mean** ±**SD**			**Mean difference [95% CI]**		
				***p*** **-value, effect size**		
	**ACLd**	**ACLr**	**Control**	**ACLd vs. ACLr**	**ACLd vs. Control**	**ACLr vs. Control**
**Positive work contribution (%)**					
Hip	55 ± 12	58 ± 10	47 ± 8	2.5 [−2.6, 7.6] *p* = 0.298, *d* = 0.32	7.8 [−0.4, 16.1] *p* = 0.063, *d* = 0.76	**10.4 [3.2, 17.5]** ***p*** **= 0.006**, ***d*** **= 1.16**
Knee	24 ± 10	23 ± 11	34 ± 7	−1.0 [−5.0, 3.0] *p* = 0.592, *d* =−0.16	**−10.1 [−16.8**, **−3.4]** ***p*** **= 0.005**, ***d*** **= −1.20**	**−11.1 [−18.3,−3.9]** ***p*** **= 0.004**, ***d*** **=−1.22**
Ankle	22 ± 8	20 ± 7	19 ± 7	−1.5 [−6.5, 3.5] *p* = 0.512, *d* =−0.20	2.3 [−3.7, 8.2] *p* = 0.441, *d* = 0.30	0.7 [−4.7, 6.2] *p* = 0.782, *d* = 0.11
**Negative work contribution (%)**					
Hip	54 ± 9	57 ± 4	50 ± 8	3.4 [−2.1, 8.9] *p* = 0.200, *d* = 0.39	3.4 [−3.3, 10.2] *p* = 0.306, *d* = 0.41	**6.8 [1.6, 12.0]** ***p*** **= 0.013**, ***d*** **= 1.04**
Knee	28 ± 8	27 ± 5	35 ± 8	−1.5 [−8.3, 5.3] *p* = 0.633, *d* =−0.14	**−6.8 [−13.3**, **−0.2]** ***p*** **= 0.043**, ***d*** **= −0.83**	**−8.3 [−13.9,−2.6]** ***p*** **= 0.006**, ***d*** **=−1.16**
Ankle	18 ± 5	16 ± 5	15 ± 4	−1.9 [−5.1, 1.3] *p* = 0.226, *d* =0.37	3.3 [−0.4, 7.0] *p* = 0.079, *d* = 0.71	1.4 [−2.2, 5.0] *p* = 0.417, *d* = 0.32

^a^P-value from a non-parametric test.

The bold values indicate the comparisons with statistically significant differences between the groups.

### Association between knee extensor strength and movement strategy

**Figure 6:**
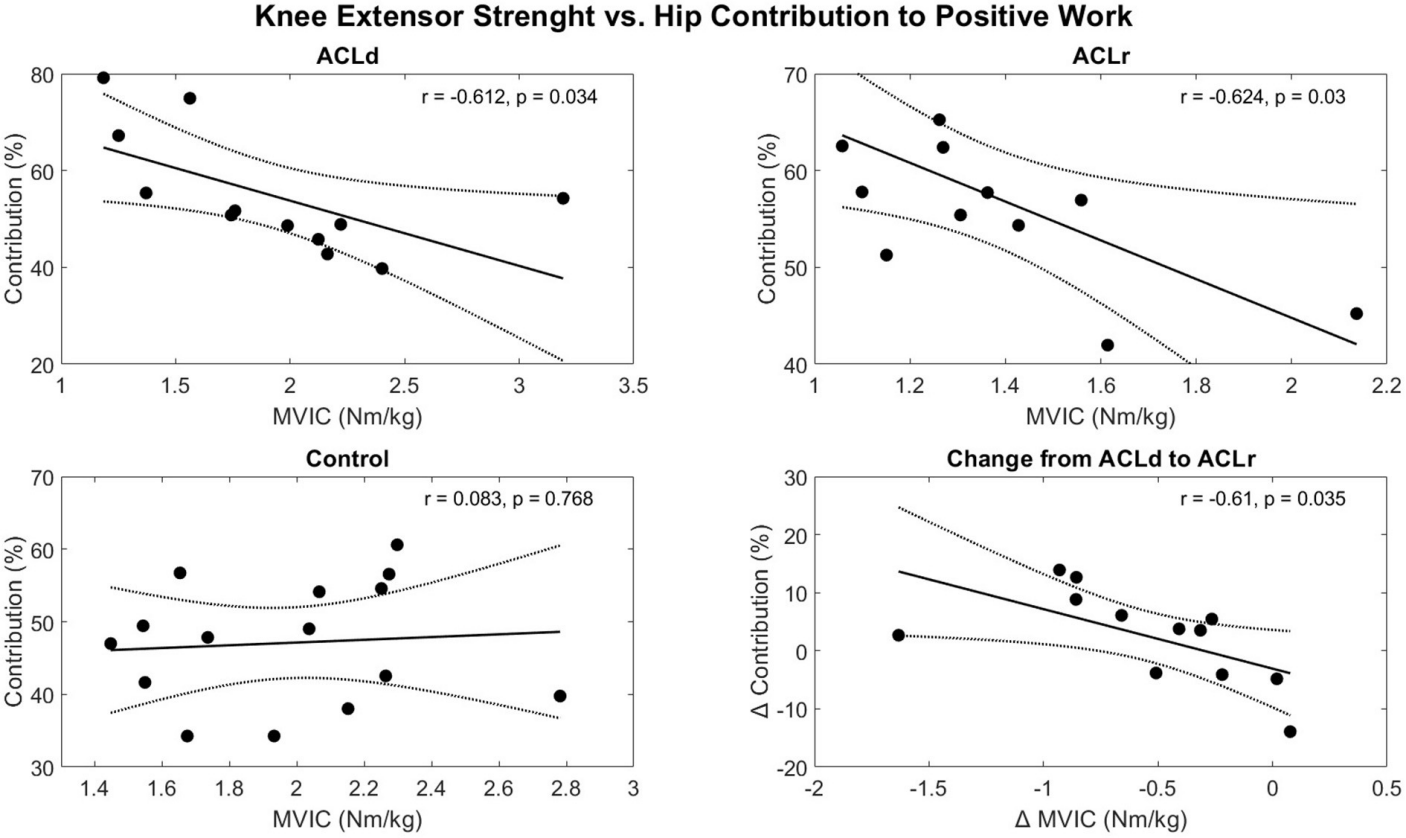
Correlations between the knee extensor strength and hip contribution to positive work performed during the forward lunge. A significant negative correlation was observed between knee extensor strength and hip contribution to the positive work both before and after ACL reconstruction. In addition, the change in knee extensor strength and the change in hip contribution were correlated in the patients with an ACL injury. The solid line represents the best fit line and the dotted lines the 95% confidence bounds of the fit.

Knee extensor strength was not statistically significantly associated with knee joint contribution to positive or negative work in ACLd, ACLr or Control. Neither was the change in knee extensor strength associated with the change in knee contribution to the work in the patients with an ACL injury (Supplementary Figures 2, 3). However, knee extensor strength was negatively correlated with the hip contribution to positive work in the ACLd (r = 0.612, *p* = 0.034) and ACLr (r = 0.624, *p* = 0.030) groups but not in Control (r = 0.083, *p* = 0.768). For the hip contribution to negative work, a significant correlation was observed in the ACLd (r = 0.658, *p* = 0.020, Supplementary Figure 4). In addition, there was a significant negative correlation between the change in knee extensor strength and the change in hip contribution to positive (r = 0.610, *p* = 0.035, Figure 6) and negative work (r = 0.627, *p* = 0.029, Supplementary Figure 4) in the patients with an ACL injury.

## Discussion

In this study, knee function was assessed longitudinally, before and after ACL reconstruction, and compared to healthy controls, by self-reported knee function, functional performance, muscle strength testing, movement quality (subjective and objective) and lower limb biomechanical analysis. To further explain previous findings the additional data examined here included muscle strength testing and full lower limb biomechanics and movement quality analysis of the forward lunge performance. The results of the study did not support our hypothesis that the patients with an ACL injury would be weaker than the controls before the reconstruction. Instead, it was observed that the patients had relative comparable muscle strength before the reconstruction, but knee extensor and flexor muscle strength declined from pre to post ACL reconstruction showing weaker muscles compared to controls at the later time point. This may be explained by the fact that patients were instructed to perform muscle strengthening exercises before the surgery. The results supported our hypothesis that patients with an ACL injury will increase the mechanical output of hip extensors to compensate for the deficit in knee extensors. This was observed in the peak hip extensor moment and peak positive hip joint power in the forward lunge. It was additionally observed that the patients with an ACL injury had an altered movement strategy compared to controls when performing the forward lunge. The altered movement strategy presented as a movement in which the same kinematic pattern was achieved with an altered contribution of hip and knee extensor muscles to the mechanical work performed. This alteration was present both before and after the reconstruction. Interestingly, the altered movement pattern did not show a tendency to be normalized from pre to post reconstruction. Instead, the alteration was strengthened. Finally, the objectively measured movement quality showed improvements from pre to post ACL reconstruction as per our hypothesis but this could not be detected in the subjective evaluation. The frequency of participants rated in the worst movement quality category showed a tendency to be larger in the patients with ACL injury compared to healthy controls, as per our expectation, although a definite conclusion could not be drawn.

### The discrepancy between different measures of knee function

Self-reported knee function and functional performance (forward lunge ground contact time) improved in the ACL reconstructed patients from pre to post reconstruction. In addition, the functional performance improved from pre to post reconstruction and was comparable to the healthy controls at the later time point. In addition, an experienced clinician was not able to detect substantially different movement qualities between the patients with an ACL injury and controls neither before nor after the reconstruction in the subjective evaluation of the forward lunge movement. These subjective results (PROMS and movement quality) convey a different message than muscle strength testing and detailed biomechanical evaluation. These objective measures showed a decrease in knee extensor muscle strength in the ACL injured patients from pre to post reconstruction, a persistent deficit in knee extensor mechanical output and an altered contribution of the lower limb joints to the mechanical work performed during the forward lunge compared to healthy controls. This difference in observations between the subjective and objective measurements suggests that the subjective measures that are easily accessible to clinicians and are most often used in clinical practice may be unable to display underlying deficits in this patient group.

### Subjective and objective evaluation of movement quality

The frequency of reported movement quality issues, assessed by the clinician, was not clearly greater in the patients with an ACL injury compared to controls nor there was a clear pattern of improvement in the movement quality within the patients with an ACL injury. On the other hand, the objective evaluation showed a statistically significant reduction in the mediolateral knee movement (knee “wobble”) and the lateral movement of the hip suggesting an improved control of the lower limb during the activity from pre to post ACL reconstruction. The difference between the clinicians' observations and objective analysis is probably not related to the reliability of the assessment. A clear consistency in the assessments was observed and a previous investigation reported that dynamic knee alignment can be evaluated reliably by an experienced clinician in forward lunge (25). Most likely the visual evaluation is not sensitive enough for detecting the subtle changes in movement quality that the objective analysis was able to detect. For example, the average reduction in the lateral hip movement was around 1 cm. It is unlikely that a clinician could detect such a small change in the movement. We still anticipated that a clinician could see features in the movement pattern indicative of poor movement control or qualities that are difficult to parametrise and are a composite of many features of the movement. Therefore, we expected that the subjective evaluation could have shown improvements in the movement quality from pre to post reconstruction. The findings based on objective movement quality assessment encourage the use of objective motion-capture-based movement quality assessment in future research studies. In most clinical settings, performing laboratory-based biomechanical analysis is not feasible but automated quantitative analysis of video images enabled by computer vision may improve sensitivity to detect subtle improvements in movement quality and control in the future (26).

### Movement strategy

We observed an altered movement strategy in the ACL injured patients in which the contribution of knee joint work on the total lower limb work was lower compared to the control group. This was observed for both the negative (eccentric work) and the positive (concentric work) work phase. This observation is consistent with recent work by Kotsifaki et al. (12–14) who reported that patients with an ACL injury have reduced knee extensor and increased hip extensor work contribution in horizontal and vertical jumps compared to uninjured contralateral limb and healthy controls.

The reason for the altered movement strategy is not clear. One explanation for the altered joint contributions could be that the patients were insecure about loading the knee joint. In support of this idea, kinesiophobia has been observed after ACL injury and it is associated with altered lower limb mechanics in drop landing (27) and gait (28). However, kinesiophobia was not assessed in the current study and therefore a conclusion on the role it played cannot be made. Another possible explanation is that the altered movement strategy is related to the functional deficit in the knee extensor muscles as a negative correlation between the knee extensor muscle strength and hip joint contribution to work was observed. Additionally, the change in knee extensor muscle strength correlated with the change in hip joint contribution giving more confidence in the potential causal connection between the variables.

The clinical implications of the altered movement strategy are currently unknown. Kinematically, the forward lunge movement was performed comparably between the patients with an ACL injury and healthy controls but the contribution of the lower limb joints to the work performed during the movement differed and peak knee extensor moment was smaller in the ACL injured patients. The altered movement strategy could serve as a mechanism to unload ACL in functional tasks. Quadriceps muscle forces increase ACL loading while hamstrings can act to reduce these loads (29). ACL reconstruction with a hamstring graft, as performed for the majority of the patients included in the current study, is known to result in deficits in hamstring muscle strength (30). Thus, the movement strategy in which lower knee extensor moments, and therefore lower quadriceps forces, are utilized may help with unloading ACL and can be especially important for ACL reconstructed patients with potentially reduced capacity to unload ACL *via* hamstring muscle forces. The potential implications of the altered movement strategy warrant further investigation for both short-term (e.g., reinjury risk) and long-term (e.g., osteoarthritis) health effects.

### Limitations

The small sample size is a limitation of the study. This prevented us from using statistical methods to compare the subjective evaluation of movement quality between the groups and meant that only the effects of large magnitude could be detected with statistical inferences. However, we considered that the effects should be large to provide clinically meaningful information. Therefore, our study can inform which measures can give clinically valuable information. In addition to the small sample size, the sample was highly variable regarding time from injury to pre reconstruction test, physical activities performed by the participants and likely in pre reconstruction rehabilitation protocols and concomitant knee injuries although we do not have detailed information on those. The heterogeneity of the sample should be kept in mind when interpreting the results meaning that the results cannot be generalized to all patients with an ACL injury. Another limitation of our study is that we chose not to perform an analysis on interlimb differences, as done in previous investigations (12–14), but rather to investigate longitudinal changes over time and compare the results to a group of healthy individuals. The reason for this was that the uninjured limb would be unable to provide an unbiased comparison due to potentially decreasing capacity in the uninjured limb during the follow-up or adaptations in the movement strategy to preserve symmetry between the limbs. This could lead to observations of between-limb symmetry that can be falsely interpreted as an indication of returned lower limb function or improved lower limb function over time (31–33). A potential problem with the comparison to the healthy control group instead of to the uninjured limb is the increased interlimb variability. Finally, additional measures that were missing from the current investigation such as electromyography to assess the magnitude of agonist-antagonist co-contraction, interpolated twitch technique to assess voluntary activation capacity and muscle strength testing in isokinetic dynamometer to give insight on joint torque-angle relationship and torque-angular velocity relationship would help to mechanistically explain the observations made in this study.

### Future directions

In the current study, we followed the patients with ACL injury until ~11 months post ACL reconstruction and found developing deficits in knee extensor and flexor muscle strength that were also reflected in an altered movement strategy. A meta-analysis of the current literature points out that these deficits are present in this patient population even after 24 months from injury or reconstruction (1). Hence, the deficits seem to be long-lasting. The literature suggests a major neural component (1, 34) in addition to the loss of muscle size (35) as an explanation for the reduced muscle force output. Restoring the muscle function is a major challenge potentially complicated by the loss of the sensory role of ACL (36–38) that could play a role in the development of the observed persistent neural deficits. Future efforts should be placed on improving the rehabilitation procedures to restore muscle function after an ACL injury.

Detailed biomechanical analysis using a motion capture system is not currently feasible for routine clinical evaluation of patients with ACL injury due to the cost of devices and their operation including the need for trained staff and the time it takes to perform the assessment and generate feedback. However, the results of the current study suggest that such analysis is needed to detect the altered movement strategy utilized by patients with an ACL injury as those kinetic alterations were not reflected in the kinematics of the movement. It may also be necessary for detecting subtle improvements in lower limb movement quality and control that cannot be detected by visual observations. The information provided by the biomechanical analysis may be useful for return-to-sport decision-making and for informing the rehabilitation process but required more accessible technologies for wide clinical adaption. More accessible technologies are currently being developed, [see e.g., (39)] but future work is needed to improve and validate the technologies to support their transfer to clinical practice.

### Conclusions

It was observed that patients with an ACL injury can perform a functional movement with a similar kinematic pattern, in regards to both joint angles and movement speed, and without observable movement quality issues although having a significant deficit in knee extensor and flexor muscle function. This is possible due to a compensatory increase in the mechanical output of hip extensor muscles. The compensatory strategy was found to be associated with knee extensor muscle strength. As other clinically feasible assessments such as self-reported knee function, functional performance, and visual evaluation of movement quality and control may mask potential deficits in knee function monitoring and comparing muscle strength with measures from a matching reference population may be a clinically feasible method for detecting the deficits in knee function. In conclusion, the current study calls for awareness of compensatory movement strategies, not observable from lower limb kinematics, that may hide deficits in knee function after an ACL injury. The findings in this explorative study should be verified in a larger study.

## Data availability statement

The original contributions presented in the study are included in the article/Supplementary material, further inquiries can be directed to the corresponding author.

## Ethics statement

The studies involving human participants were reviewed and approved by Ethics Committee for the Capital Region of Denmark (H-3-2013-126) and the University of Ottawa Ethics Board (H06-14-27). The patients/participants provided their written informed consent to participate in this study.

## Author contributions

TA, DB, and MK designed the study. TA, KS, TF, and DB conducted the experiments. JS, MS, and LS wrote the analysis code. JS, MS, TA, CB, and LS analyzed and interpreted the data. LS wrote the initial manuscript draft. CB, JS, MS, KS, TF, DB, MK, and TA revised the manuscript. DB, MK, LS, and TA contributed to funding acquisition and project administration. All the authors read and approved the final manuscript.

## Funding

This project was supported by: Canadian Institutes for Health Research, Natural Science and Engineering Research Council, The Aase and Ejnar Danielsens Fund, the Danish Rheumatism Association grant R130-A3612, the Lundbeck Foundation grant R143-2013-12690, Innovation Fund Denmark 9088-00006B-under the frame of ERA PerMed and Academy of Finland 332915. The funders had no role in study design, data collection and analysis, decision to publish, or preparation of the manuscript.

## Conflict of interest

The authors declare that the research was conducted in the absence of any commercial or financial relationships that could be construed as a potential conflict of interest.

## Publisher's note

All claims expressed in this article are solely those of the authors and do not necessarily represent those of their affiliated organizations, or those of the publisher, the editors and the reviewers. Any product that may be evaluated in this article, or claim that may be made by its manufacturer, is not guaranteed or endorsed by the publisher.
