# Supplementary material for: Altered movement strategy during functional movement after an ACL injury, despite ACL reconstruction
Source: Front Sports Act Living. 2022 Oct 4;4:994139. doi: 10.3389/fspor.2022.994139 (PMC9576999; doi:10.3389/fspor.2022.994139)
Supplement: Supplementary file 2 [file Data_Sheet_1.docx]

Supplementary Material

# Results for the subjective evaluation of movement quality

A clinical assessment of movement quality was performed by an experienced physiotherapist based on animations of the lower limb skeletal movement. The evaluation results are presented below. For the overall movement quality rating the rating “Not OK” means that the movement quality raises concern regarding the potential increased risk of a knee injury.

# Supplementary Figures

**
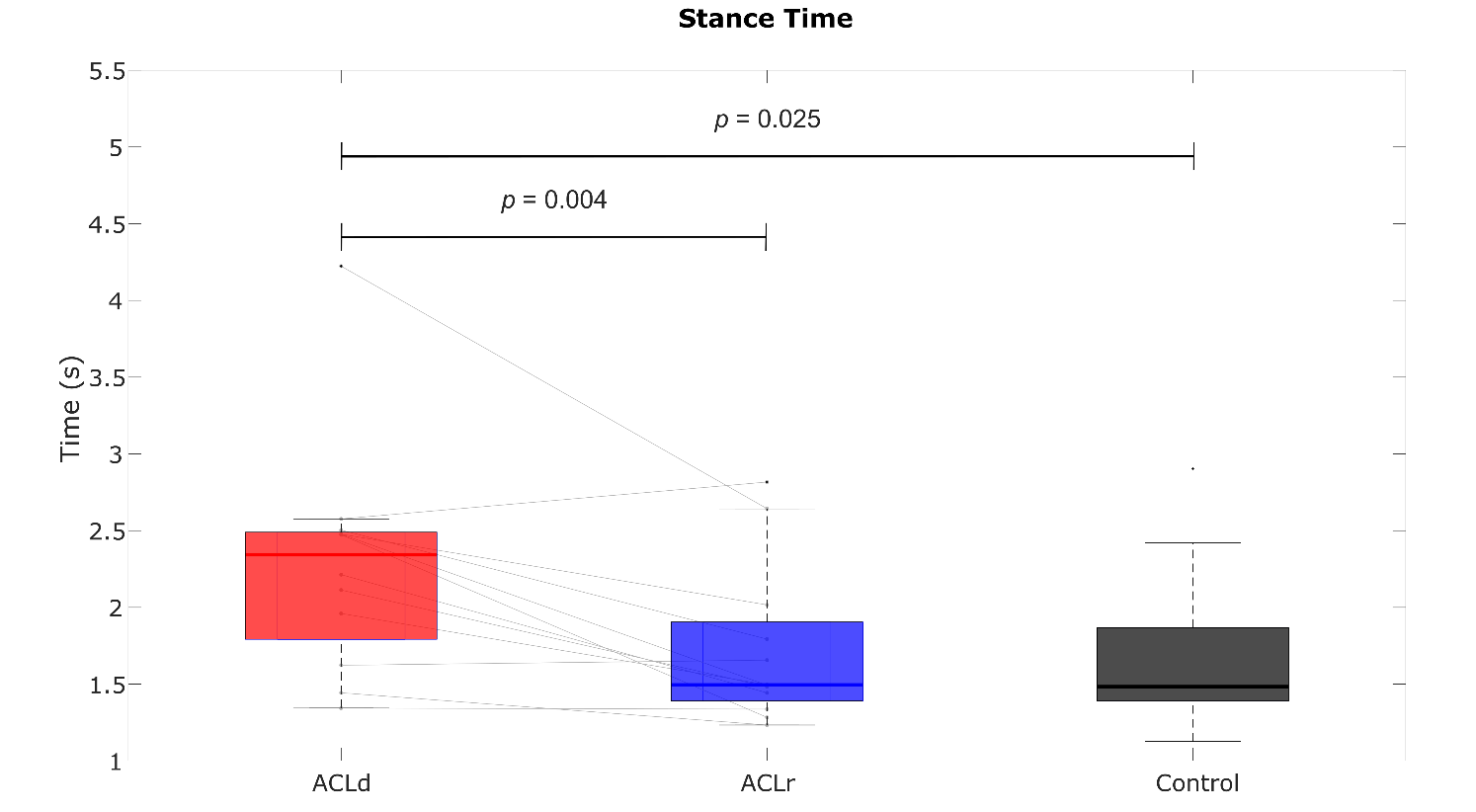
**

**Supplementary Figure S1.** The duration of the stance phase of the forward lunge movement. The time was longer for the ACL deficient patients compared to healthy controls and was reduced from pre to 10 months post ACL reconstruction.


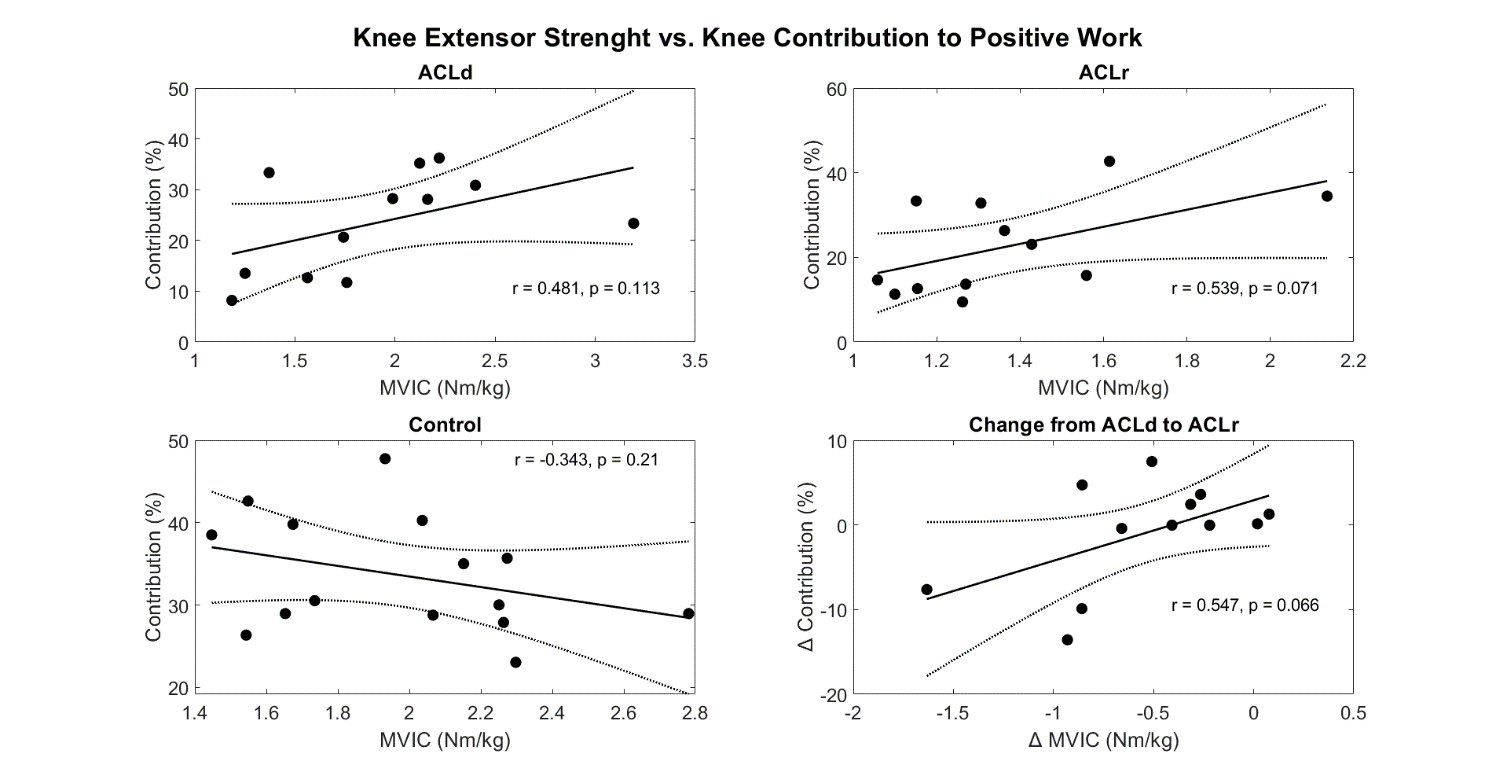


Supplementary Figure S2*.* Correlations between the knee extensor strength and knee contribution to positive work performed during the forward lunge.


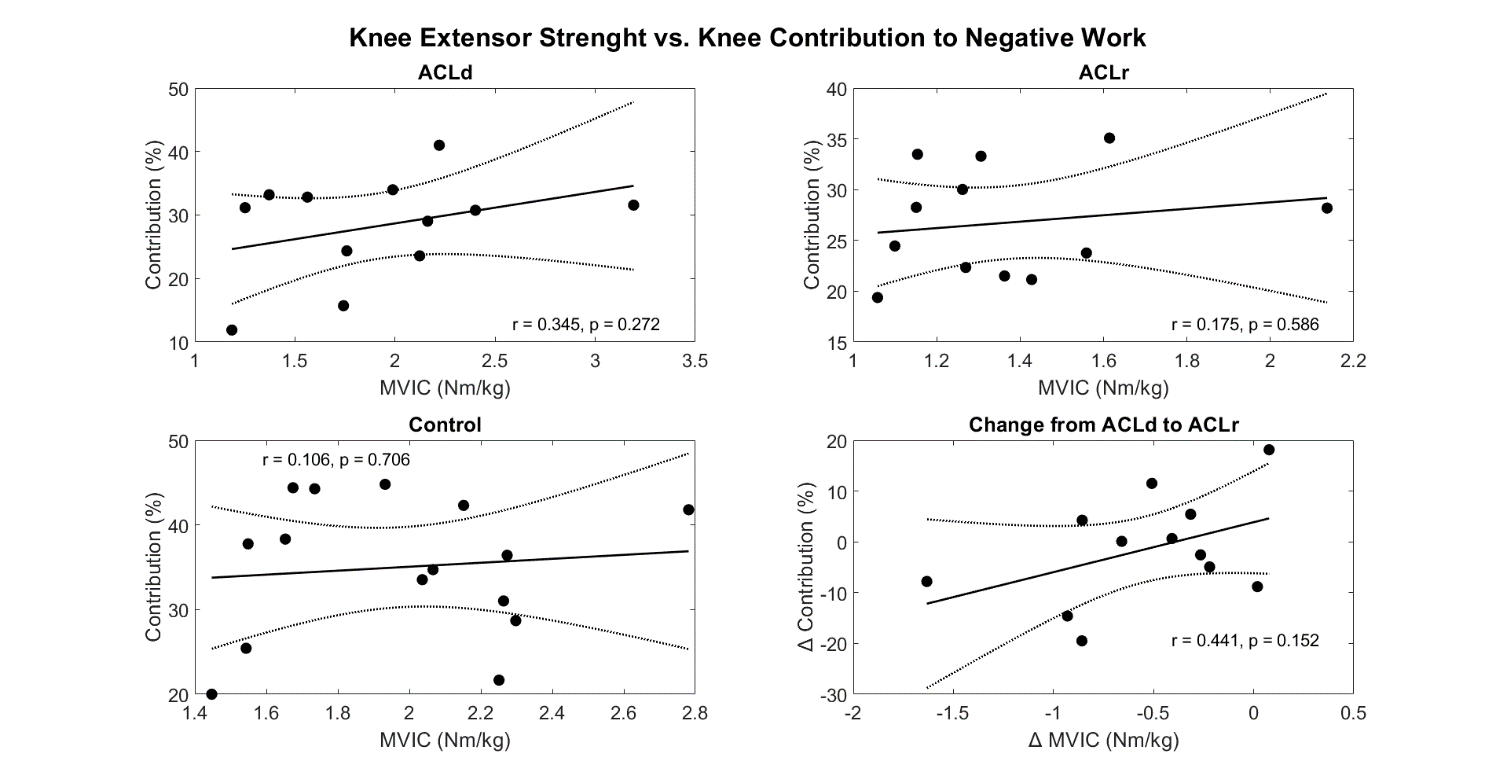


Supplementary Figure S3*.* Correlations between the knee extensor strength and knee contribution to negative work performed during the forward lunge.


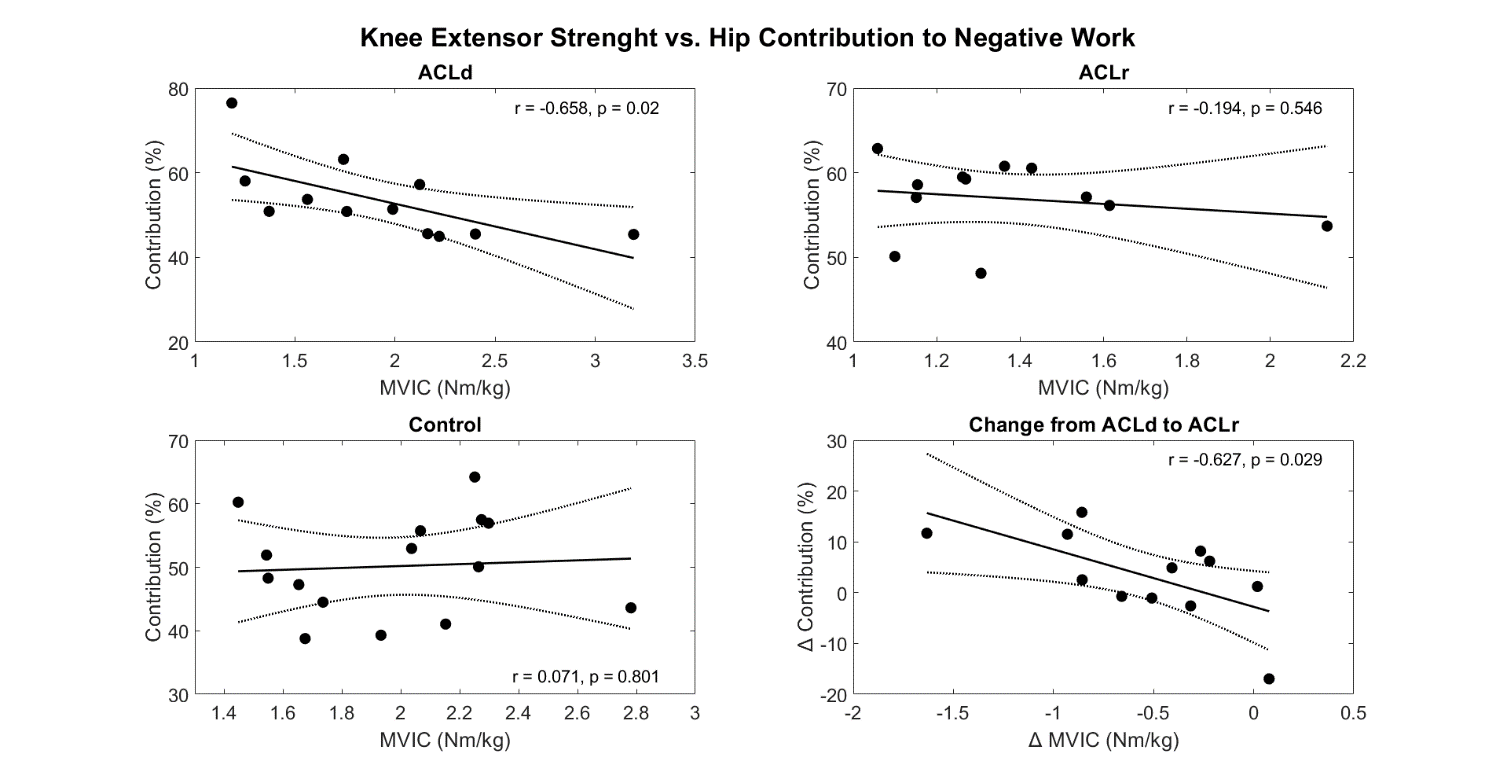


Supplementary Figure S4*.* Correlations between the knee extensor strength and hip contribution to negative work performed during the forward lunge. A significant negative correlation was observed between knee extensor strength and hip contribution to the negative work before ACL reconstruction. In addition, the change in knee extensor strength and the change in hip contribution were correlated in the patients with an ACL injury.
